# The Effect of a High-Grain Diet on the Rumen Microbiome of Goats with a Special Focus on Anaerobic Fungi

**DOI:** 10.3390/microorganisms9010157

**Published:** 2021-01-12

**Authors:** Katerina O. Fliegerova, Sabine M. Podmirseg, Julia Vinzelj, Diego J. Grilli, Simona Kvasnová, Dagmar Schierová, Hana Sechovcová, Jakub Mrázek, Giuliana Siddi, Graciela N. Arenas, Giuseppe Moniello

**Affiliations:** 1 Laboratory of Anaerobic Microbiology, Institute of Animal Physiology and Genetics, Czech Academy of Sciences, 14220 Prague, Czech Republic; kvasnova@iapg.cas.cz (S.K.); schierova@iapg.cas.cz (D.S.); sechovcova@iapg.cas.cz (H.S.); mrazek@iapg.cas.cz (J.M.); 2 Institute of Microbiology, University of Innsbruck, A-6020 Innsbruck, Austria; Sabine.Podmirseg@uibk.ac.at (S.M.P.); Julia.Vinzelj@uibk.ac.at (J.V.); 3 Área de Microbiología, Facultad de Ciencias Médicas, Universidad Nacional de Cuyo, Mendoza M5500, Argentina; diegogrilli@yahoo.com.ar (D.J.G.); gnarenas@yahoo.com.ar (G.N.A.); 4 Department of Veterinary Medicine, University of Sassari, 07100 Sassari, Italy; giuliana.siddi@gmail.com (G.S.); moniello@uniss.it (G.M.)

**Keywords:** Neocallimastigomycota, ITS1, ITS2, LSU, high-throughput sequencing, clone library, diet switch

## Abstract

This work investigated the changes of the rumen microbiome of goats switched from a forage to a concentrate diet with special attention to anaerobic fungi (AF). Female goats were fed an alfalfa hay (AH) diet (0% grain; *n* = 4) for 20 days and were then abruptly shifted to a high-grain (HG) diet (40% corn grain, 60% AH; *n* = 4) and treated for another 10 days. Rumen content samples were collected from the cannulated animals at the end of each diet period (day 20 and 30). The microbiome structure was studied using high-throughput sequencing for bacteria, archaea (16S rRNA gene) and fungi (ITS2), accompanied by qPCR for each group. To further elucidate unclassified AF, clone library analyses were performed on the ITS1 spacer region. Rumen pH was significantly lower in HG diet fed goats, but did not induce subacute ruminal acidosis. HG diet altered prokaryotic communities, with a significant increase of Bacteroidetes and a decrease of Firmicutes. On the genus level *Prevotella* 1 was significantly boosted. *Methanobrevibacter* and *Methanosphaera* were the most abundant archaea regardless of the diet and HG induced a significant augmentation of unclassified Thermoplasmatales. For anaerobic fungi, HG triggered a considerable rise in *Feramyces* observed with both ITS markers, while a decline of *Tahromyces* was detected by ITS2 and decrease of *Joblinomyces* by ITS1 only. The uncultured BlackRhino group revealed by ITS1 and further elucidated in one sample by LSU analysis, formed a considerable part of the AF community of goats fed both diets. Results strongly indicate that the rumen ecosystem still acts as a source for novel microorganisms and unexplored microbial interactions and that initial rumen microbiota of the host animal considerably influences the reaction pattern upon diet change.

## 1. Introduction

The worldwide number of goats (*Capra aegagrus* f. *hircus*) is constantly increasing, together with the role of this livestock in food production [1]. According to American Goat Federation (americangoatfederation.org) there are approximately 450 million goats around the whole world. In some regions, especially the arid zones, goats are outnumbering cattle and are frequently more numerous than sheep, thus having a strong influence on the socio-economic life of human populations. The increase in demand for milk and meat production has induced intensive production systems, where goats are often fed relatively high-grain (HG) diets to achieve the maximum performance. This feeding pattern is able to saturate energy and protein requirements of the animal for maintenance and growth, but on the other hand is known to induce changes in the rumen microbiota [2,3,4,5] and often causes metabolic disorders [6,7,8].

The effect of HG diets on the health and diversity of the rumen microbiome of goats has received considerable attention in the past decade. Concentrate-rich diets induce an increase of the lactic acid and/or butyrate production/accumulation [9,10,11] and a decrease of the rumen pH that in turn results in higher proportion of Firmicutes [11,12,13]. At the genus level the reduction of *Fibrobacter* [14,15] and fibrolytic bacteria sensitive to low pH [9] has been described, while the genera *Lactobacillus*, *Enterococcus*, and *Succiniclasticum* were increased in goats fed a HG diet [10,11,14]. On the other hand, the abundance of some species, especially *Prevotella*, but also *Butyrivibrio*, is highly variable, giving inconsistent results [10,12,14,16,17,18], indicating the genetic diversity, diet specificity, and functional diversity of some rumen microbial groups [11].

All these studies show considerable influence of HG diet on the bacterial population, while information on eukaryotic members of the goat rumen ecosystem is very scarce. Metzler-Zebeli et al. [14] microscopically evaluated ciliate protozoa in the rumen fluid of goats fed different proportion of barley grain (0%, 30%, and 60%) showing that the increasing dietary grain level linearly increased the number of entodiniomorphids, whereas that of holotrich ciliates did not change. Similarly, Liu et al. [10] observed a higher protozoa number with the increase of grain (50–80%) in goat diets. However, at the onset of sub-acute ruminal acidosis (SARA), the protozoan population declined dramatically. Although it is known that anaerobic fungi (AF) (phylum Neocallimastigomycota, class Neocallimastigomycetes, order Neocallimastigales, family Neocallimastigaceae [19]), represent a major monophyletic group of symbiotic microorganisms, occupying the forestomach of ruminants and the hindgut of nonruminant herbivores, their diversity in relation to the animals’ diet in goats has remained unexplored. Together with bacteria and protozoa, AF contribute to the hydrolysis of dietary fibers resulting in the production of end fermentation products (volatile fatty acids), which are utilized by the host animals as nutrient source [20]. An impressive array of enzymes necessary for plant cell-wall degradation enables AF to break down plant cell walls, access fermentable substrates, degrade and weaken plant tissue, and reduce the size of the dietary particles [21,22,23]. Moreover, the fungal rhizoidal systems, which can be highly branched and invasive, are able to penetrate deeply into the plant stems and can aid to mechanically decompose the plant substrates [24]. Due to the capability of AF to colonize the sturdy plant structures and recalcitrant components, such as the sclerenchym and vascular system, it can be expected that Neocallimastigomycota play an especially important role in goats, which are used to live in harsh arid environments and are consuming low-quality feed rich in fiber, lignin, and tannins [25,26].

Although the diversity of goat rumen fungi has not been comprehensively studied, the recently described genus *Liebetanzomyces* [27] and the latest described genera *Agriosomyces*, *Capellomyces*, and *Joblinomyces* [28] were isolated from the feces of goats, pointing at a yet untapped source of novel fungal species associated with this host animal. The number of cultured AF has been enlarged up to 19 genera including as of yet *Neocallimastix*, *Piromyces*, *Orpinomyces*, *Anaeromyces*, *Caecomyces*, *Cyllamyces*, *Buwchfawromyces*, *Oontomyces*, *Liebetanzomyces*, *Pecoramyces*, *Feramyces*, *Ghazallomyces*, *Aklioshbomyces*, *Agriosomyces*, *Capellomyces*, *Joblinomyces*, *Khoyollomyces*, *Tahromyces*, and *Aestipascuomyces*, the last ten of which were described only very recently [28,29,30,31]. Among these known genera, the species of *Piromyces spiralis* [32], *Neocallimastix californiae* [33], *Anaeromyces contortus* [34], and the novel species of *Agriosomyces longus*, *Capellomyces foraminis*, *Capellomyces elongates*, and *Joblinomyces apicalis* [28] have been isolated from the rumen fluid and feces of domesticated and/or wild goats.

Further literature on goat AF is quite limited, involving the measurement of the hydrolytic activities of *Neocallimastix* sp. from rumen of native Indian and Korean goats [35,36,37] and the isolation of AF and associated methanogens of *Neocallimastix frontalis*, and *Caecomyces communis* strains from feces of the Alpine ibex (Capra ibex) [38]. However, a pyrosequencing study by Liggenstoffer et al. [39], did not detect any sequences of *Neocallimastix* and *Caecomyces* in the feces of domesticated goat, but determined *Anaeromyces* (48%), *Piromyces* (33%) and an uncultured group AL5 (20%) as prevalent genera. The cultured representative of the group AL5 was isolated recently from the feces of the domesticated goat and sheep and described as *Joblinomyces* [28,40]. A study by Kok et al. [41] evaluated the ability of goat rumen fungal populations to adapt to the dietary high concentrations of condensed tannins by real-time PCR. Finally, the sequences of xylan degrading enzymes of GH family 11 related to xylanases from *Neocallimastix* and *Orpinomyces* were detected in the rumen fluid of grazing goats [42].

To the best of our knowledge, there is no report on the influence of high grain diet compared to high-fiber diet on the diversity and abundance of the prokaryotic, and also the anaerobic fungal rumen microbiome of goat. A higher proportion of concentrate is one of the feeding management strategies used in intensive caprine production systems, however rational use of grain as the main energy resource has to be consistent not only with productivity but also with animal health. This study was, therefore, undertaken to evaluate both feeding regimes and their effect on the bacterial, archaeal, and fungal population in rumen fluid of fistulated goats. In addition, the role of a host-animal-specific AF population was evaluated. A culture-independent, high-throughput sequencing (HTS) approach together with qPCR analyses were chosen to analyse these microbial groups within rumen fluid of Creole goats during an alfalfa hay (AH) and high grain (HG) feeding phase. Furthermore, AF diversity was more deeply investigated by clone libraries targeting the ITS1 gene sequences.

## 2. Materials and Methods

### 2.1. Animals and Diet

This study was conducted at the Scientific and Technologic Center located in Mendoza City, Argentina. The experiment was permitted by The Committee for the Care and Use of Animals in Research of the University of Juan Agustín Maza, Mendoza, Argentina (Approval No. 102/2013) and performed in accordance with the Guide for Care and Use of Agricultural Animals in Research and Teaching of the Federal Animal Science Society [43]. Four Creole goats (meat breed, 5-year-old, BW: 40 ± 5 kg, never having an offspring) fitted with a rumen fistula were used in this study. The animals housed in pens were fed on the AH diet for a period of 20 days and were then abruptly shifted to a mixed HG diet consisting of 60% alfalfa hay and 40% corn grain for another 10 days. The diets, formulated to meet the animals’ nutrient requirements, as defined by the National Research Council [44], were composed of 1.96 or 2.33 Mcal of metabolizable energy (ME) kg^−1^ of dry matter with forage-to-concentrate ratios of 100:0 (AH diet) and 60:40 (HG diet), respectively. The goats were fed simultaneously once daily in the morning, had free access to water, and had not been co-housed with any other animals.

### 2.2. Nutritional Composition of AH and HG Diets

Samples of alfalfa hay (*Medicago sativa*) and corn grains were dried at 70 °C for 72 h, reduced to a particle size of less than 1 mm, and subjected to chemical analyses (Table 1): dry matter (DM), crude protein (CP), starch content, neutral detergent fiber (NDF), acid detergent fiber (ADF) [45]; acid detergent lignin (ADL) [46]; hemicellulose [47]; and cellulose [48]. Additionally, the ME content was estimated according to Aguilera [49].

### 2.3. Sample Collection and DNA Extraction

The rumen content (200 mL) was collected 5 h after feeding at the end of the AH diet period (day 20) and ten days after the change of the diet to HG (day 30). Simultaneously, the ruminal pH was measured with a glass electrode and clinical criteria for acute or subacute ruminal acidosis, such as decreased intake, weakness, and abdominal pain were used to evaluate the impact of the abrupt change of the diet on the animal health. A representative aliquot of each sample was freeze-dried and shipped to the Institute of Animal Physiology and Genetics, Czech Academy of Sciences (Prague, Czech Republic) for the DNA isolation and subsequent analyses. The genomic DNA was extracted using the method of Yu and Morrison [50]. The concentration and purity of the extracted nucleic acids were checked using a NanoDrop 2000c UV-Vis spectrophotometer (Thermo Scientific, Waltham, MA, USA). DNA extracts were stored at −20 °C until use.

### 2.4. Characterization of Goat Rumen Microbiome by High-Throughput Sequencing

The DNA extracts were subjected to an amplicon sequencing approach using the Illumina MiSeq chemistry v3 (300 bp paired-end) and targeting specific marker genes of the archaeal, bacterial and fungal population within the rumen liquid under AH and HG diet (*n* = 4). The sequencing was performed by Microsynth AG (Balgach, Switzerland) using the following primers: 515f (GTGCCAGCMGCCGCGGTAA) and 806r (GGACTACHVGGGTWTCTAA) targeting the V4 region of the archaeal and bacterial 16S rRNA gene [51] and ITS3 (GCATCGATGAAGAACGCAGC) and ITS4 (TCCTCCGCTTATTGATATGC) targeting the eukaryotic ITS2 region [52]. Both PCR steps (first stage of library preparation and barcoding) were performed with the KAPA Hifi Hotstart polymerase system. The conditions for the library generation for both V4 and ITS2 were: initial denaturation at 95 °C for 3 min, followed by 30 cycles (V4) or 35 cycles (ITS2) of denaturation at 98 °C for 20 s, annealing at 56 °C for 30 s and elongation at 72 °C for 30 s. The runs were completed with a final elongation step at 72 °C for 5 min. Both libraries were generated in separate reaction tubes and equimolarly pooled for subsequent barcoding.

The CoMA pipeline [53] was used for the data processing of obtained raw V4 reads. In brief, paired-end reads were merged, barcodes and primers trimmed and sequences with an average quality score of ≥25 and a median length of 253 bp of the 16S rRNA gene (V4) were kept for further analyses. For archaea and bacteria, the alignment and taxonomic affiliation of sequences were undertaken at a 97% similarity level using the blast algorithm and the SILVA SSU (release 128) and Greengenes (release 13_5) as primary and backup databases, respectively. Datasets were subsampled according to the sample with the lowest read number, singletons and doubletons removed and only OTUs (operational taxonomic units) retained that could be detected in at least two samples. For a general overview at phylum or genus-level, only OTUs with a minimum abundance of ≥1% are shown.

For analysis of the fungal community, an amplicon sequence variance (ASV)-based strategy was chosen over an OTU-based one since ASVs are more suitable to recover fungal community composition [54]. Fungal sequences (ITS2) were analyzed using the DADA2 algorithm [55], basically following the DADA2 ITS Pipeline Workflow (1.8) available on the official DADA2 homepage. In short, primers have been removed from the demultiplexed reads using cutadapt [56], then N-filtering and trimming has been done using the filterAndTrim function of the DADA2 package with the following parameters enforced: maxN = 0, maxEE = c(2,2), truncQ = 2, truncLen = c(245,200), rm.phix = TRUE, compress = TRUE). For taxonomic assignment the assignTaxonomy tool of DADA2 and the UNITE database v. 04.02.2020 [57] enriched with the ITS2 sequences of the novel AF genera [28] was used. Further analysis was done in R, mostly following the example workflow for DADA2 downstream analysis posted by Happy Belly Bioinformatics [58]. For better visualization of the diversity patterns, all ASVs that were not present at least three times in at least two samples were discarded. Changes in community composition at phylum and, if applicable, down to species level were normalized and analyzed using unfiltered ASV count data with DESeq2 [59] adapted for microbial count data [60].

SRA files of the 16S rRNA (V4) and ITS2 HTS approach were deposited to the NCBI database under BioProject PRJNA602992 and BioSamples SAMN13915736-SAMN13915751 with their respective SRA numbers SRR10961333-SRR10961348.

### 2.5. Characterization of Anaerobic Fungi Population Using Clone Libraries

The amplification of the anaerobic fungal ITS1 region from each sample was carried out with the combination of the fungal universal ITS1 forward primer (CTTGGTCATTTAGAGGAAGTA) [61] and the Neocallimastigomycetes-specific 5.8S rRNA gene reverse primer (GTGCAATATGCGTTCGAAGATT) [62] as described previously by Mura et al. [63]. The PCR reaction (50 µL) was performed with a PPP Master Mix kit (Top-Bio, Vestec, Czech Republic) and 0.3 μM of each primer using the following thermal cycling conditions: 33 cycles of denaturation at 94 °C for 1 min, annealing at 58 °C for 30 s and extension at 72 °C for 45 s with an initial cycle of 94 °C for 4 min and final cycle of 72 °C for 2 min. Each sample was PCR amplified in quadruplicate, pooled, and verified by agarose gel electrophoresis. The pooled PCR amplicons of the correct length (approximately 350 bp) were excised from an agarose gel with a sterile scalpel blade, purified, and concentrated using a QIAquick Gel Extraction Kit (Qiagen, Hilden, Germany). For sample G3AH the ribosomal large subunit 28S rRNA (LSU) was also amplified in quadruplicate using Neocallimastigomycetes-specific primer set GGNL1F (5′-CATAGAGGGTGAGAATCCCGTA-3′) and GGNL4R (5′-TCAACATCCTAAGCGTAGGTA-3′) as described previously by Nagler et al. [64]. The rationale for this analysis is explained in the results section.

The TOPO^®^ TA Cloning^®^ Kit for Sequencing (Life Technologies, USA) was used for the preparation of the ITS1 (and, for sample G3AH, LSU) clone libraries for each sample as described by Mura et al. [63]. Plasmid DNA was isolated from 413 randomly selected ITS1 clones (in average 51 clones per library) and 35 LSU clones of *E. coli* using a GenElute™ HP Plasmid Miniprep Kit (Sigma-Aldrich, St. Louis, MI, USA) and sent for Sanger sequencing using the M13F priming sites within the vector pCR4 (SEQme, Dobris, Czech Republic). The quality of reads was manually verified, the vector contamination was removed and chimeras were identified by examining the multiple sequence alignment using bioinformatics software Geneious 10.3.1. (https://www.geneious.com). Unambiguous sequence data were processed in QIIME version 1.8 [65] by BLAST against ITS1 reference database of AF [66] updated with sequences of novel species. The 413 cloned sequences with taxonomic assignment from the eight libraries were concatenated into a single fasta file, and a sequence map (seqs_otus.txt) was manually built for input into the script “make_otu_table.py”. The resulting relative abundance table was summarized at the clade level. The analysis of LSU sequence data was carried out using BLAST against GenBank database. The ITS1 nucleotide sequences generated from rumen samples of goats by clone library approach have been deposited to the NCBI database under the following accession numbers: MH038788—MH039011 (AH diet) and MH039012—MH039235 (HG diet). The LSU sequences of the sample G3AH have been deposited to the NCBI database under the accession numbers MW364027—MW364061.

### 2.6. qPCR Analysis

The quantification of ITS1 gene copy numbers was performed using the MX 3005P QPCR System (Stratagene) and EliZyme Green MIX AddROX (Elizabeth Pharmacon). The same ITS1 primer pair and cycling conditions as described above for clone library preparation were used. The melting curve analysis with an interval from 58 to 95 °C was conducted to verify the amplification specificity. The standard curve (slope −3.258, correlation coefficient (*R*^2^) 0.991) was prepared from a 10-fold dilution series of a pCR4-TOPO plasmid containing the ITS1 region of *Anaeromyces mucronatus* BF2 (NCBI Accession No. MK418978). The quantitation was linear over seven orders of magnitude (10^2^ to 10^8^ gene copies μL^−1^). All samples and negative controls without a DNA template were analyzed in three replicates. Total bacteria and total archaea were quantified on a Rotor-Gene Q real-time cycler using the SensiFast SYBR^®^ Hi-Rox chemistry (Bioline, London, UK). Freshly prepared ten-fold dilutions (10^7^ to 10^2^) of *Nitrosomonas europea* (DSMZ 21879) and *Methanobrevibacter formicium* (DSMZ 1535) stock DNA served as reference together with 1:10 dilutions of sample extracts, all analyzed in technical duplicates. For archaea the primer pair Parch519f/Arch915r [67,68] at a final concentration of 400 nM and for bacteria 515f/806r [51] at a concentration of 250 nM were used. Cycling consisted of 95 °C for 10 min, followed by 40 cycles of 95 °C for 20 s, 57 °C or 50°C for 20 s (archaea or bacteria, respectively), and 72 °C for 30 s each. A final melt curve (65 to 90 °C) with an increment of 0.25 °C was added after each run. R^2^-values of standard curves were above 0.999. The calculation of 16S rRNA copy numbers was performed with the Rotor-Gene Series Software (2.3.1) and for all investigated groups copy numbers were calculated per g of fresh matter (FM) rumen sample.

### 2.7. Statistical Analysis

The differences between the two diets were tested for significance using a paired *t*-test. In case of violation of normal distribution (Shapiro–Wilk test), the Wilcoxon singed rank test was used instead. Pearson correlation analysis was used to evaluate the (inter) dependencies of species data or correlation with surveyed diet characteristics. Significant differences were declared at the *p* < 0.05 level. Pearson correlation analyses and their visualizations were done in R using the packages corrplot, and lares. A quantitative profile of bacteria, archaea, and anaerobic fungi was calculated for each sample, using the product of obtained gene copy numbers and relative abundance of each clade as given by high-throughput sequencing.

## 3. Results

### 3.1. Influence of Diet on Ruminal pH

The corn grain supplementation significantly increased the starch content (*p* < 0.05) in HG diet (Table 1), which induced acidification of the rumen environment. The pH of the rumen fluids of HG-fed goats (6.49 ± 0.10) was significantly lower (*p* < 0.05) compared to AH-fed goats (6.82 ± 0.12). However, ruminal pH values of animals fed the HG diet still ranged in physiological levels. None of the goats showed any clinical or sub-clinical signs of acidosis indicating that the abrupt transition of animals from AH to HG diet did not cause either acute or subacute ruminal acidosis.

### 3.2. High-Throughput Sequencing of the 16S rRNA Gene V4 Region and the ITS2 Region

For V4 amplicons, a total of 34,939 reads for all eight goat rumen samples passed quality filtering of the original 71,533 reads obtained by high-throughput sequencing (HTS). While the average number of reads per sample was 4367 ± 1800, subsampling was performed at 1646 reads per sample. Based on the rarefaction curves, this allowed for a satisfactory sequencing depth (Supplementary Figure S1) to determine the core microbiome. Analysis of the V4 region revealed a bacterial and archaeal sequence distribution of 96.3 to 3.7%. Altogether 605 and 8 OTUs were detected for bacteria and archaea, respectively. OTUs that were not present at least three times in at least two samples were discarded. The OTU abundance was evenly distributed amongst all samples in the V4 dataset (273 ± 22) and the Shannon index (average 6.81 ± 0.2) indicated that all samples showed equally high diversity (Supplementary Figure S2).

After filtering, denoizing, merging, and chimera removal within the DADA2 pipeline, 322,326 ITS2 reads (60.8% of input reads) remained for taxonomic assignment. Then, 350 ASVs were identified, of which 132 ASVs remained after filtering of low abundance groups. On average across all samples, most of the filtered reads were assigned to Neocallimastigomycota (67.78 ± 3.53%), followed by Ascomycota (19.64 ± 2.15%), Basidiomycota (8.17 ± 0.98%), unidentified Fungi (4.40 ± 0.33%), and Mucoromycota (0.01 ± 0.00%).

#### 3.2.1. Bacterial Community Composition

**Figure 1:**
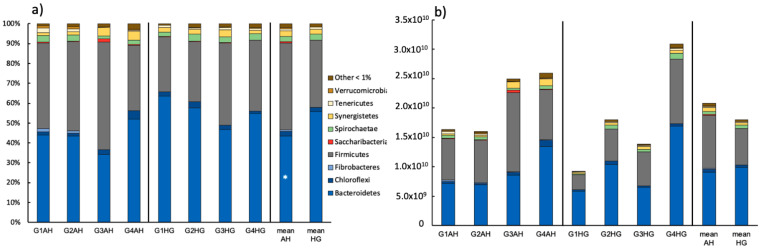
(**a**) Relative ruminal bacterial community composition of goats fed an AH diet (left) or HG diet (middle) and mean values of both treatments (right), respectively (phylum-level). Results are based on amplicon sequencing of the V4 region of the 16S rRNA gene and phyla ≥ 1% abundances are depicted; (**b**) Absolute bacterial community composition based on relative abundance and total bacterial number as retrieved via qPCR. Significant changes between treatments are indicated by an asterisk.

The bulk bacterial community consisted of the phyla Bacteroidetes (43.4 ± 7.24% AH; 55.7 ± 6.94% HG) and Firmicutes (43.8 ± 8.74% AH; 33.7 ± 6.09% HG) and some minor populations of Synergistetes, Spirochaetae, Chloroflexi, Tenericutes, Fibrobacteres, Saccharibacteria, and Verrucomicrobia in descending order (Figure 1a). All these groups except for Bacteroidetes and Spirochaetae were decreasing in relative abundance following the diet switch of AH to HG and the relative increase of the key group of Bacteroidetes was significant. At genus level a significant increase of *Prevotella* 1 could be observed (Supplementary Table S1). Other major genera found were *Prevotella*, members of the Rikenellaceae RG9 gut cluster, an uncultured rumen bacterium cluster, Christensenellaceae R-7, *Butyrivibrio* and *Succiniclasticum* to name only a few. Looking at the bacterial abundance a slight overall decrease of 16S rRNA copies from 2.08 × 10^10^ ± 5.36 × 10^9^ (AH) to 1.8 × 10^10^ ± 9.29 × 10^9^ (HG) was observed after diet change (Figure 1b). However, this went along with very individual responses of the bacterial abundance in each goat (Supplementary Table S2).

#### 3.2.2. Archaeal Community Composition

**Figure 2:**
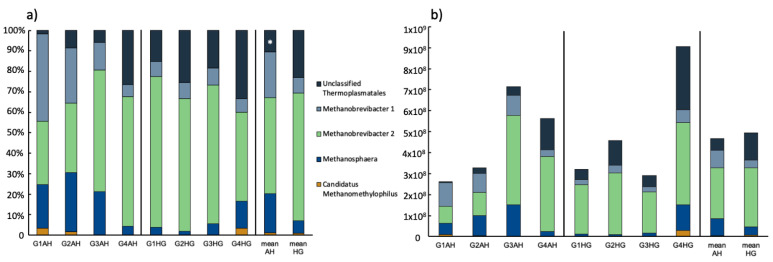
(**a**) Relative ruminal archaeal community composition of goats fed an AH diet (left) or HG diet (middle) and mean values of both treatments (right), respectively. Results are based on amplicon sequencing of the V4 region of the 16S rRNA gene and genera ≥ 1% abundance are depicted; (**b**) absolute archaeal community composition based on relative abundance and total archaeal number as retrieved via qPCR. Significant changes between treatments are indicated by an asterisk.

After 20 days of AH diet, the archaeal rumen population was basically composed of *Methanobrevibacter* (two different OTUs; 69.3 ± 5.86%), *Methanosphaera* (18.9 ± 10.3%) and a yet unclassified Thermoplasmatales archaeon (Figure 2; Supplementary Table S1). Only in two animals (G1AH, G2AH) Candidatus *Methanomethylophilus* could be detected. All these genera were distributed at varying abundance, whereas G1AH and G2AH seemed to have a more similar archaeal rumen microbiome (Figure 2a). Following the diet switch to HG, a disappearance of Candidatus *Methanomethylophilus* was observed in the two latter mentioned animals, this genus emerged, however, at little abundance (3.3%) in G4HG (Figure 2a). There was no significant change in relative abundance for the two genera *Methanobrevibacter* and *Methanosphaera* upon diet change, however, it induced a significant increase in the unclassified Thermoplasmatales group from 10.6 ± 11% to 23.1 ± 8.11% (*p* < 0.01). Regarding archaeal abundance, a slight increase of 16S rRNA copies from 4.66 × 10^8^ ± 2.1 × 10^8^ (AH) to 4.93 × 10^8^ ± 2.84 × 10^8^ (HG) was observed after diet change. Archaeal rumen population rose in three goats and decreased in one animal (Figure 2b, Supplementary Table S2).

#### 3.2.3. Fungal Community Composition

**Figure 3:**
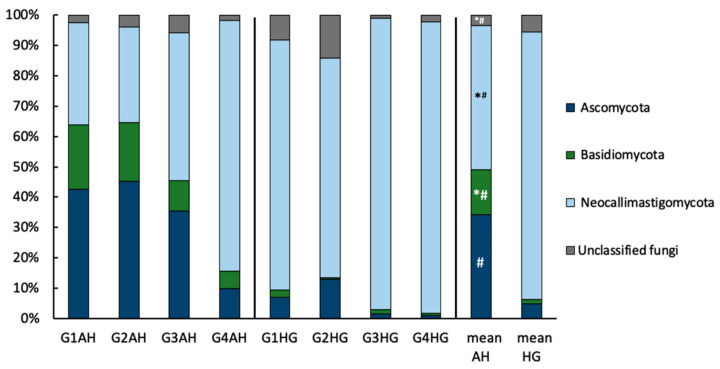
Relative ruminal, fungal community composition at phylum-level of goats fed an AH diet (left) or HG diet (middle) and mean values of both treatments (right), respectively. Results are based on amplicon sequencing of the ITS2 region and phyla ≥ 1% abundance are depicted. Significant changes between treatments are indicated by an asterisk (*t*-test on relative genus-abundance data) or hash (based on DESeq2 analysis of ASV raw data).

The fungal rumen microbiome after 20 days of AH diet was comprised to the largest extent of Neocallimastigomycota (47.7 ± 18.0%), Ascomycota (34.1 ± 17.7%), and Basidiomycota (14.9 ± 8.8%) (Figure 3). The community composition shifted more towards Neocallimastigomycota (88.3 ± 18.9%) after the change to HG diet. The increase in Neocallimastigomycota on HG diet was accompanied by a drop in Ascomycota (4.9 ± 3.4%), and Basidiomycota (1.3 ± 0.8%). Under both diet regimes, a small community of unclassified fungi could also be detected (3.3 ± 1.2% and 5.5 ± 3.7%, for AH and HG diet, respectively). Additionally, under AH diet a minor population of Mucoromycota was detected in two animals (Supplementary Table S1). While the individual community composition differed from animal to animal, the trend of community changes at phylum level upon diet switch were the same for all animals. DESeq2 analysis identified the drop in Ascomycota and Basidiomycota, as well as the increase in Neocallimastigomycota and unclassified Fungi upon diet change to be significant (*p* < 0.05; Supplementary Figure S3). It further identified 22 ASVs out of 350 ASVs to exhibit significant changes upon diet switch.

Among Ascomycota, the genus of *Aspergillus* formed a major part of the community in goats on AH diet (15% of filtered reads). However, the steep drop in relative abundance upon diet switch (0.27% of filtered reads in goats on HG diet) was not identified as significant by DESeq2 analysis. Instead, six other Ascomycota genera (e.g., *Myrothecium, Lectera*) significantly dropped, and only two Ascomycota genera (*Ascochyta,* and *Neosetophoma*) increased in relative abundance upon diet change.

### 3.3. Community Composition of Neocallimastigomycota Based on HTS of the ITS2

**Figure 4:**
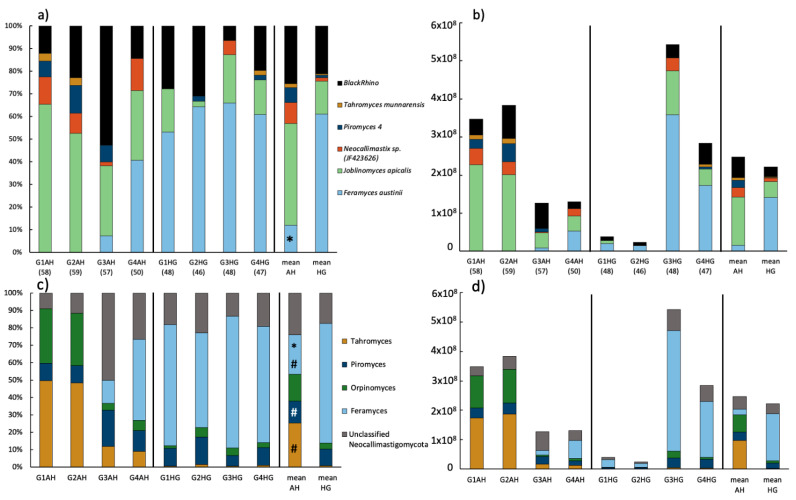
Neocallimastigomycota community composition of rumen samples of goats fed either AH or HG and mean values of both treatments: (**a**) relative and (**b**) absolute abundance of anaerobic fungi as detected by ITS1 clone library approach. The number of analyzed clones is given in brackets. (**c**) Relative and (**d**) absolute abundance of anaerobic fungi as detected by amplicon sequencing of the ITS2 region. Absolute abundance is based on qPCR results. Significant changes between treatments are indicated by an asterisk (*t*-test on relative genus-abundance data) or hash (based on DESeq2 analysis of ASV raw data).

At the genus level, 12 ASVs across 4 genera of Neocallimastigomycota were identified by the DADA2 algorithm; one additional ASV could not be identified beyond belonging to Neocallimastigaceae. While individual differences between animals were less prominent at phylum level, at genus/species level those differences became clearer. For example, the Neocallimastigomycota community of goats G1 and G2 on AH diet mainly consisted of *Tahromyces*, and *Orpinomyces*, while goat G3 was dominated by unclassified Neocallimastigaceae and goat G4 was dominated by *Feramyces* (Figure 4c; Supplementary Table S1). Generally, it seemed that the microbiome of goats G1 and G2 was fairly similar compared to goats G3 and G4. The influence of the individual microbiome composition was especially obvious in the qPCR results: goats G1 and G2 reacted to the diet switch with a decrease by the factor of ten for total Neocallimastigomycota counts (from 3.66 × 10^8^ to 3.1 × 10^7^), while in goats G3 and G4 the numbers trippled from 1.29 × 10^8^ up to 4.13 × 10^8^. The overall trend was a drop in Neocallimastigomycota numbers by 10.1% upon diet change (Figure 4d). Furthermore, the increase of starch content in the diet led to a Neocallimastigomycota community conversion across all goats (Figure 4c). There was a significant increase in *Feramyces* (acc. DESeq2 analysis) after the diet switch. The rise in *Feramyces* in goats G1 and G2 (from 0 to 69.4% and 54.3%, respectively) was accompanied by a drop in *Orpinomyces* and *Tahromyces* and an increase in *Piromyces* and unclassified Neocallimastigacaea. For goat G4, the further increase of *Feramyces* (from 46.7 to 66.8%) led to a drop in all other identified genera (Figure 4c, Supplementary Table S1).

Broken down to ASV level, 7 ASVs assigned to Neocallimastigaceae showed significant changes upon diet switch (Supplementary Figure S3). Interestingly, while all three ASVs identified as *Feramyces* showed a significant increase, the other four ASVs (two *Tahromyces* and two *Piromyces*) behaved inconsistently (one of each increased; the other decreased).

### 3.4. Community Composition of Neocallimastigomycota Based on ITS1 Clone Libraries

The relatively high proportion of unclassified anaerobic fungi obtained by ITS2 sequencing, especially in the samples G3AH, G1HG, and G2HG, induced the further analysis of AF diversity using ITS1 barcode marker via a clone library approach. All of the 413 screened ITS1 fragments from eight clone libraries were taxonomically assigned within the phylum Neocallimastigomycota (Figure 4a). Overall, already cultured genera represented the 67.1% majority of sequences and were taxonomically assigned to *Feramyces* (34.4%)*, Joblinomyces* (31.5%)*,* and *Tahromyces* (1.2%). A portion of 27.3% of sequences were represented by genus-level groups called BlackRhino (23.2%) and *Piromyces* 4 (4.1%) (as defined by Koetschan et al. by [66]) which are currently without cultured representatives. A portion of 5.6% of sequences was assigned to JF423626, which is the accession number to a cultured strain of *Neocallimastix frontalis* NZRold-05 [69] (Figure 4a). In the ITS1-sequence, structure-based analysis of AF by Koetschan et al. [66]; however, this sequence clustered into the genus *Orpinomyces*. Thus, in the ITS1 reference database this specific AF strain is listed with the accession number only.

The shift from AH to HG diet promoted a change in relative abundances of fungal taxa (Figure 4a, Supplementary Table S3). The mean results per treatment showed that *Joblinomyces* was the dominant genus in AH-fed goats (44.9 ± 5.5%), followed by the BlackRhino group (25.5 ± 18.7%), *Feramyces* (12 ± 19.5%), sp. JF463626 (9.3 ± 5.5%), *Piromyces* 4 (6.6 ± 5.1%), and *Tahromyces* (1.7 ± 1.9%). The relative abundance of *Feramyces* quintupled in the HG-fed goats up to 61.1 ± 5.7%, while all the other fungal groups were decreased, in particular *Joblinomyces* dropped by a factor of three (14.5 ± 8.5%) (Figure 4a, Supplementary Table S3). The individual response of animals to the diet shift is evident, especially when ITS1 qPCR data are considered (Figure 4b). Goats G1 and G2 with initially no detectable sequences of *Feramyces*, but with high relative abundance of *Joblinomyces* (Figure 4a), exhibited a suppression of AF concentration (Figure 4b), decreased abundance of *Joblinomyces* and appearance of *Feramyces* under HG diet. In contrast, the HG diet induced higher AF concentration in the goats G3 and G4 (Figure 4b), characterized mainly by the enhancement of relative abundance of *Feramyces* (Figure 4a). The overall ruminal population of Neocallimastigomycota reacted with a slight drop from 2.47 × 10^8^ ± 1.38 × 10^8^ (AH) to 2.22 × 10^8^ ± 2.45 × 10^8^ (HG) upon diet change (Figure 4b).

### 3.5. Community Composition of Neocallimastigomycota in Sample G3AH Based on LSU Clone Library

**Figure 5:**
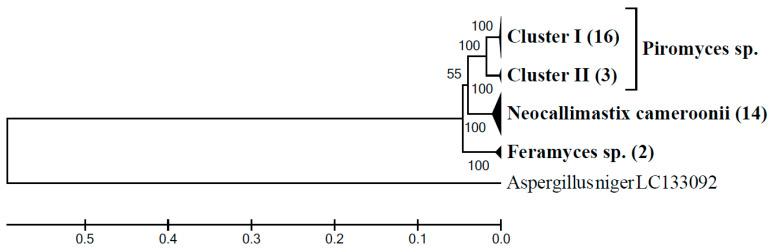
Phylogenetic relationships of anaerobic fungal LSU gene sequences generated from the sample G3AH inferred using the UPGMA method with bootstrap values from 1000 replications. The analysis involved a total of 45 nucleotide sequences (35 sequences generated from the G3AH sample, 9 sequences of the cultured genera of anaerobic fungi (*Piromyces* sp. MK775335, KY368606, and KY368602, *N. camerooni* NG060329, KR920745, and MG992493, *F. austinii* MG584215, MG584217, and MG605676) plus 1 outgroup sequence. An uncompressed phylogenetic tree is shown in (Supplementary Material Figure S4).

In the rumen of goat G3 on AH diet the considerable 52.7% of the ITS1 sequences belonged to the monophyletic BlackRhino clade (Figure 4a). In order to get more information about taxonomic position of this uncultured clade, this sample was also analyzed using an LSU based clone library. Tree topology showed that the LSU sequences obtained (*n* = 35) formed four distinct clusters (Figure 5). The clade of *Piromyces* sp. included two clusters. Clones representing cluster I (*n* = 16) had the highest sequence identity (99.3–99.6%) to a sequence of a cultured *Piromyces* sp. strain SFH682 (MK775335) isolated from sheep feces. Clones of the sister cluster II (*n* = 3) had the highest sequence identity (99.5–99.8%) to a cultured *Piromyces* sp. strain CN12 (KY368606) isolated from goat rumen. Fourteen other clones had the highest sequence identity (98.3–99.8%) to *Neocallimastix* cf. *cameroonii* strain G3 (MG992493) isolated from sheep feces. Only two clones clustered with *Feramyces* sp. showing 99.5% sequence identity to *Feramyces austinii* strain F2a (MG605676) isolated from Barbary sheep feces. Interestingly, NCBI BLAST searching of region of local similarity between BlackRhino ITS1 (both sequences generated in this study and sequences from reference ITS1 database) and cultured species resulted in high sequence identity (99.1%) of 332 long ITS1 spacer to *Piromyces* sp. strain SFH682 (MK775329), which is the same strain identified here by LSU sequences analysis.

### 3.6. Correlation Analyses

**Figure 6:**
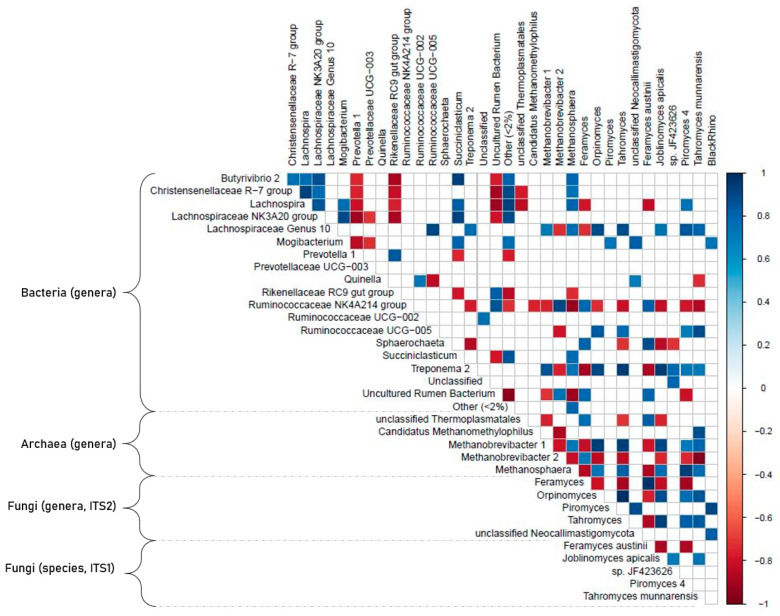
Correlogram of Pearson’s correlation pairs. Based on relative abundance data from the V4 HTS (OTU based), ITS2 HTS (ASV-based), and ITS1 clone library approaches. Only pairs with *p* < 0.05 and −0.7 < r < 0.7 are shown.

A total of 71 parameters (diet characteristics, HTS results for archaea, bacteria, total fungi and Neocallimastigomycota, and clone library results) were subjected to a correlation analysis. Out of the 317 pairs that showed significant correlation (*p* < 0.05) at high levels (i.e., −0.8 > Pearson’s r > 0.8), the top parameters with the most connected partners were: Fibrobacteres (18 connections), *Treponema* 2 (15), Verrucomicrobia (14), Basidiomycota (13), *Lachnospiraceae* Genus 10 (13), *Methanobrevibacter* 1 (12), *Lachnospira* (11), and Ascomycota (11). For more details please see the correlation table and related figures (Figure 6, Supplementary Table S4). Here we focus on the strongest correlation pairs and interactions of Neocallimastigomycota with other microbial groups or physico-chemical parameters.

Out of the dietary metadata, only starch content showed correlations with the phylogenetic data. It positively correlated with *Feramyces austinii* (ITS1 clone library data; r +0.88), *Feramyces* (ITS2 HTS data; r +0.86), and with the bacterial groups of Ruminococcaceae NK4A214 (V4 HTS data; r +0.85) and Sphaerochaeta (V4 HTS data; r +0.96). Neocallimastigomycota, in general, negatively correlated with Ascomycota (r −0.99) and Basidiomycota (r −0.93). At higher phylogenetic resolution, however, *Tahromyces* (ITS2), *Orpinomyces* (ITS2), *Piromyces 4* (ITS1), and *Joblinomyces apicalis* (ITS1) showed a high positive correlation with Ascomycota. Within archaea, *Methanobrevibacter* 1 was negatively correlated with *Feramyces* (both ITS1, and ITS2 data), but positively correlated to *Tahromyces* (ITS1 and ITS2), *Orpinomyces* (ITS2), and *Joblinomyces apicalis* (ITS1). In general, there were three different clusters of Neocallimastigomycota correlations: the first cluster was dominated by *Feramyces* (ITS1 and ITS2 data) and positively correlated with high starch content, the bacterial phylum of Bacteroidetes (V4 HTS data; r +0.72), and the archaeal group of unclassified Thermoplasmatales (r +0.76). A second cluster with a more diverse community that consisted of *Orpinomyces* (ITS2), *Joblinomyces* (ITS1), *Piromyces 4* (ITS1), and *Tahromyces* (ITS1 and ITS2) and positively correlated with the bacterial phyla Fibrobacteres, and Verrucomicrobia and with the archaeal genera of *Methanosphaera* and *Methanobrevibacter* 1. Furthermore, the third cluster consisted of unclassified Neocallimastigomycota genera (ITS2), *Piromyces* (ITS2), and BlackRhino (ITS1). This last cluster generally shared little connections to the rest; only the BlackRhino group correlated positively with *Methanosphaera* and the bacterial genus of *Lachnospira* NK3A20, and negatively with Rikenellaceae RC9 Gut Group and a cluster of uncultured rumen bacteria.

## 4. Discussion

The animals’ diet is the major determinant of ruminal microbial composition [70,71] and it depends highly on the geographical location and the type of production system. Creole goats in Argentina are mostly kept extensively with continuous grazing on rangelands and little or no feed supplementation [72,73]. Economic aspects of meat and milk production, however, necessitate alternative feeding systems and it is a common strategy to add substantial portions of grains to diets to saturate the animals’ nutritional requirement. It is well known that feeding excessive amounts of non-structural carbohydrates results in subacute ruminal acidosis (SARA) [7]. The development of SARA seems to be dependent on the ratio of forage and grain with no signs of this metabolic disorder in goats fed up to 30% of barley grain [74]. In our study, 40% of corn grain in the diet has not induced sub-clinical signs of acidosis, despite pH-values being significantly lower in HG fed goats. However, in any of studied goats ruminal pH did not drop below 5.6, which is a value established by Khafipour et al. [75] to consider a SARA in individual animals. A study by Lee et al. [76] indicates, that goats can be more resistant to ruminal acidosis compared to cattle. This could be related to some goat-specific evolutionary adaptations of the digestive system such as large salivary glands, the large absorption area of rumen epithelium, the ability to rapidly change the rumen volume in response to environmental alterations, efficient recycling of urea, efficient use of water, and the ability to reduce their metabolism rate [25].

The impact of diet changes on the microbiome of goats, however, still remains unclear. The number of studies dealing with this topic has been increased recently [77,78], mainly with emphasis on bacterial diversity, while the response of anaerobic fungi to dietary treatment is less studied and in the goat model essentially unexplored.

### 4.1. Prokaryotic Response on Diet Shift

The HTS data of this study revealed the equal proportion of the two main bacterial phyla Firmicutes and Bacteroidetes in AH fed goats, while the transition from feeding on hay to corn-containing diet (40%) resulted in a significant increase of Bacteroidetes. This result is not consistent with previous reports describing that the high, readily fermented carbohydrates in HG diets lead to a higher proportion of Firmicutes [11,12,13,18,79]. In fact, in our study, Firmicutes even decreased by approximately 25% in relative abundance. The content of grain in the mentioned studies was, however, at least 65%, which might explain the detected differences. Interestingly, when comparing our results on the mature rumen microbiome of goats with the study of Wang et al. [78], Bacteroidetes were in the same range; however, Proteobacteria were clearly less abundant in our study (always below 1% compared to ≥10%). Firmicutes, in contrast, remained at higher levels in our study, even after the abundance drop that accompanied the change to HG diet. These discrepancies might be strongly influenced by the specific diet composition: the diet in the study of Wang et al. [78] was composed of a more diverse mixture (alfalfa 34.8%, rice straw 35.2%, corn 11.5%, soybean meal 5%, wheat bran 12%, salt, and mineral nutrients).

The methanogenic community composition revealed in this study corresponds well with published data [80,81,82] showing *Methanobrevibacter* and *Methanosphaera* as the two most prevalent genera in adult goats. The high incidence of Candidatus *Methanomethylophilus* described by Wang et al. [81], however, could not be confirmed in our study, as we detected this species in low amounts in three of the tested animals only. Furthermore, we report here for the first time a high abundance of an unclassified Thermoplasmatales group (two OTUs) in goat rumen (especially in HG fed goats), indicating that the rumen ecosystem still acts as a source for novel microorganisms and unexplored microbial interactions. Indeed, correlation analyses on all investigated parameters revealed that this group of unclassified Thermoplasmatales is positively connected with *Feramyces* (ITS1 and ITS2 data), and negatively connected to Ascomycota. Still, it remains unclear if this indicates a direct interaction of *Feramyces* and the unclassified Thermoplasmatales group, or if this co-increase under HG diet is triggered by other environmental conditions or other microbial guilds. Generally, groups such as *Methanobrevibacter* sp., *Methanobacterium* sp., and *Methanosphaera* have already been identified as the main methanogens associated with anaerobic fungi [38,83,84]. Looking at the key prokaryotic genera, similar members were detected for goats as described by Kumar et al. [83], mentioning *Prevotella*, Lachnospiraceae, *Methanobrevibacter,* and *Methanosphaera* amongst others in the rumen of dairy cows. In addition to the before mentioned groups, Belanche and coworkers [85] further detected *Treponema* and *Ruminococcus*, which were also present in our study. The study dealt with the microbial response upon diet shift from concentrate to high-fiber diet in sheep. However, the dominant AF in that study were *Caecomyces* sp. and *Neocallimastix frontalis*, which were not detected in our survey. A major bacterial group in our study, *Prevotella*, is a common rumen bacterium that exhibits a very versatile metabolism, able to thrive on peptides, proteins, monosaccharides and plant polysaccharides [86] and thus is often unaffected by diet changes. The increase with concentrate-rich diets, as determined in our study, was also observed by others [15,70].

### 4.2. Fungal Response on Diet Shift

The diversity of total fungi observed in our work was comparable to a similar study on Holstein heifers that dealt with the rumen microbiome under varying concentrate ratios in feed [18]. This study determined Neocallimastigomycota and Ascomycota to be the dominant fungal groups, but also detected a cluster of uncultured Fungi as well as a minor population of Basidiomycota. In another multi-kingdom study investigating the sheep rumen microbiome of grazing and non-grazing (concentrate added to the feed) animals, researchers could assign up to 80% of total fungal reads to the phylum Neocallimastigomycota [85]. In that study, as well as in our work, the phylum Neocallimastigomycota was especially prevalent in goats on HG diet. However, another study on dairy cows showed opposite trends [87]. Nevertheless, all three studies found Neocallimastigomycota to be inversely related to Ascomycota. High abundance of Ascomycota described previously in AH fed animals was associated with plant pathogens that can attack grass before the harvest [88], as well as with contamination by Ascomycota mold (especially *Aspergillus* and *Fusarium*) that can occur during storage processes [89]. In the present study, however, the high abundance of Ascomycota cannot solely be explained by these phenomenons, since the change in relative abundance of *Aspergillus* upon diet switch was not significant. Whether other fungal groups, except for Neocallimastigomycota, truly play a role in the fermentative rumen system beyond being ingested together with the feed, remains to be elucidated.

For Neocallimastigomycota in particular, it is already well established that altering the substrate availability through dietary modification affects their population structure within the digestive tract. Denman et al. [90] reported a greater diversity of Neocallimastigomycota in cattle fed high-fiber diets compared to those fed high grain-based diets (70%). This trend was also found in dairy cows [83,91], steer [92], sheep [85], and now in goats (this study). Interestingly, while Denman et al. [90] described the disappearing of the genus *Neocallimastix* in grain-fed cattle, higher levels of *Neocallimastix* and *Piromyces* were observed in another cattle study [91] as well as in sheep [85]. These inconsistencies on genus level might solely be due to differences in taxonomic databases/methods used, as the phylogenetic identification based on ITS 1 or ITS 2 barcode sequences alone, still remains challenging for the phylum of Neocallimastigomycota [93]. Furthermore, some of the genera, especially *Piromyces*, are known to be polyphyletic [94]. However, it might also be partially attributed to individual differences among host animals or even differences among individuals of the same host species (as shown in our study).

*Feramyces*, found as the prevalent genus in HG fed goats regardless of the used detection method, is an anaerobic fungal genus that has only been described recently. As an uncultured environmental sequence, this clade was first described by Liggenstoffer et al. [39], later denoted as AL6 by Kittelmann et al. [69], and finally renamed by Hanafy et al. [30] to *Feramyces* based on its first cultured representatives. This fungus is characterized by a wide substrate utilization pattern, being able to metabolize substrates often reported as not supportive for growth of other AF isolates, such as galactose, fucose, arabinose, and glucuronic acids. Moreover, some *Feramyces* strains displayed faster growth and higher extent on corn stover when compared with the majority of known cultivable AF strains [28]. All these features support our observation of increased abundance of this species in HG fed animals. *Joblinomyces* and *Tahromyces* are also newly described AF; however, there are no substrate preferences described yet [28]. While *Tahromyces* was detected in low amounts in only three animals, *Joblinomyces* formed a clear majority in AH fed goats (based on ITS1 cloning results), indicating that this genus might prefer the utilization of structural polysaccharides rather than storage carbohydrates. The sp. JF423626, here prevalent in AH fed goats, is a cultured, but not yet properly identified, strain, straddled between *Neocallimastix* and *Orpinomyces* (based on the molecular biological data). Both genera belong to the best studied AF and are known for their ability to utilize a wide spectrum of substrates, such as cellulose, xylose, glucose, starch, grass, and straw [95]. The features of the potentially new genus JF423626, however, have yet to be revealed.

Of the uncultured clades identified by ITS1 analysis, the BlackRhino group also represented a considerable part of sequences in all goats regardless of the diet and can thus be regarded as core member of the goat rumen. Further investigation of this group indicated, that AF of the BlackRhino clade, considered up to now as uncultured, could be identical to the already cultured strain *Piromyces* sp. SFH682 (MK775335) isolated from sheep feces. The fact that we could not clearly identify this group highlights the necessity of a need for another phylogenetic marker system for Neocallimastigomycota. Combining our results with qPCR data illustrated a decrease of *Joblinomyces* and increase of *Feramyces* after the addition of grain to the diet and supports the findings on strain-specific fermentation of certain types of carbohydrates [96,97]. This study thus encourages an opinion of Edwards et al. [95] about the need to re-evaluate the understanding on anaerobic fungi as simple “fiber degraders” and to reconsider their supposedly limited role in the rumen of animals fed cereal-supplemented diets.

Summarizing, after comparison of existing, corroborating, but also contradicting literature, the question remains if and to which extent these disparities arise from the host animal (e.g., cattle vs. sheep vs. goat) or varying feed compositions. Nevertheless, our results suggest that the initial, animal-specific community composition has a great influence on the microbiome shift upon the diet change. This fact was especially true for the anaerobic fungi diversity during the experiment (Figure 4). Our results highlight that 40% of concentrate proportion in the feed is not inducing SARA, but it leads to a very specific rumen microbiome that seems adapted to higher starch contents.

### 4.3. Comparison of the ITS1 and ITS2 as Taxonomic Marker for AF

According to literature the internal transcribed spacer (ITS) region is recommended as the universal DNA barcode marker for fungi [40,98]. Additionally, the 28S ribosomal large subunit (28S rRNA gene (LSU); D1/D2 region) might render higher coverage of early diverging lineages, such as Neocallimastigomycota, Mucoromycota, and Chytridiomycota [98]. Comparison of ITS1 and ITS2 has caused debates on which of these two regions was more appropriate for fungal phylogenetic analyses. Some authors could not detect significant superiority of either region for the classification of fungi, but rather an under- or over-representation of some fungal groups depending on the barcoding region [99]. Other authors clearly stated an advantage of using ITS1 over ITS2, in part due to shorter sequence lengths and lower GC content [100].

Here we focused on the taxonomic affiliation of anaerobic fungi by covering the whole range of the ITS region, and thus split the analyses into two approaches ITS2 (HTS) and ITS1 (cloning library), respectively. In this way we were able to combine the interpretation and compare the suitability of either fragment. The higher availability of ITS1 as compared to ITS2 fragments in the databases is also reflected in the slightly higher diversity obtained by ITS1 cloning than ITS2 HTS. Most of the uncultured AF have only been detected as environmental nucleic acid sequences (ENAS) by ITS1 primers, which can explain the missing BlackRhino group and *Piromyces* 4 in HTS results. However, the ITS1 region has also some disadvantages, as recently shown by Edwards et al. [101] and Yang et al. [102]. A community composition analysis can result in false higher diversity due to the heterogeneity of the ITS1 region, resulting in ambiguity in the annotation of sequences. Thus, integrating secondary structure predictions has been suggested to better classify AF sequences using ITS1 [66]. According to Edwards et al. [101] the accuracy of the method, and consequently of the results, is influenced by sample community composition, and there is an evident need to develop an alternative taxonomic marker for anaerobic fungi [93].

Nevertheless, despite differences between the results from ITS1 and ITS2 analysis and the aforementioned differences within individual animals, the trend of *Feramyces* dominance after the diet switch to HG was consistent.

## 5. Conclusions

Studies on the influence of a high grain diet on the rumen microbiome are certainly of great importance in terms of finding an optimal carbohydrate-to-fiber ratio of grain containing a diet with higher energetic and nutritional quality, but still not associated with disturbances of the ruminal microbiota leading to metabolic disorders. Strategic grain supplementation and a rational use of this nutritional resource have practical agricultural relevance for animal health and productivity.

Our results attest feasibility of abrupt diet switches to high-grain in goats, even with 40% concentrate. It seems to be a very robust system, able to quickly adapt to new nutrient conditions and still maintain a healthy microbial community. The analyses of microbial diversity and interrelationships among microbial groups suggest goat rumen to be a promising source for novel archaea (uncultured Thermoplasmatales cluster) but also anaerobic fungi isolates (BlackRhino group). Furthermore, studying this habitat more closely could reveal deeper knowledge on symbiotic interactions of AF, bacteria, and archaea. Despite a very drastic change in the composition of the anaerobic fungi population, overall absolute abundance was not significantly changed after the diet switch from AH to HG.

We could observe that, in addition to the diet, the animal-specific microbiome structure also heavily influences the response pattern upon the diet change, especially among Neocallimastigomycota.

The comparison of ITS regions, ITS1 and ITS2, for anaerobic fungi phylogenetic analysis revealed no clear advantage of one over the other. Instead, our results again highlight the need to develop a robust barcoding marker for deep-branching fungal lineages such as Neocallimastigomycota.

## Figures and Tables

**Table 1:** Total daily intake and daily intake of food, nutrients and metabolizable energy (ME) of Creole goats included in the experimental period. Mean values ± SD.

	Diets	
	Alfalfa Hay	High Grain
**Total daily intake (g DM)**	1061 ± 173 ^a^	1252 ± 193 ^a^
**Daily food intake (g DM):**		
Alfalfa Hay	1061 ± 173 ^a^	900 ± 193 ^a^
Corn	-	352
**Nutrient intake (g DM):**		
CP	168 ± 27 ^a^	171 ± 30 ^a^
NDF	361 ± 59 ^a^	335 ± 65 ^a^
ADF	228 ± 37 ^a^	204 ± 41 ^a^
ADL	42 ± 7 ^a^	39 ± 7 ^a^
Hemicellulose	133 ± 22 ^a^	132 ± 24 ^a^
Cellulose	183 ± 30 ^a^	163 ± 33 ^a^
Starch	27 ± 4 ^a^	244 ± 5 ^b^
**Daily intake of ME (MCal kg DM^−1^)**	2.08 ± 0.34 ^a^	2.78 ± 0.38 ^b^

^a,b^ Values in the same row with different superscripts differ at *p* < 0.05. DM, dry matter; CP, crude protein; NDF, neutral detergent fiber; ADF, acid detergent fiber; ADL, acid detergent lignin.

## Data Availability

The experiment was performed in accordance with the Guide for Care and Use of Agricultural Animals in Research and Teaching of the Federal Animal Science Society.

## Supplementary Materials

The following are available online at https://www.mdpi.com/2076-2607/9/1/157/s1, Table S1: Taxonomic composition of goat rumen liquid under different diets (AH at d20 and HG at d30) based on DNA amplicon sequencing targeting the ITS2 region (Fungi and Neocallimastigomycota) and the 16S rRNA gene V4 region (Archaea and Bacteria). Read abundance (for V4 at ≥ 1%) is given at phylum- and genus-level. Additionally, for each treatment mean values ± SD are shown (*n* = 4). Anaerobic fungi results highlighted in grey. Table S2: Abundance of anaerobic fungi, bacteria and archaea in rumen liquid samples of goats fed different diets (AH at d20 and HG at d30) determined by qPCR based on ITS1 and 16S rRNA copy number per g fresh matter (FM). Mean values of each sample (technical replicates *n* = 3 for anaerobic fungi and *n* = 2 for bacteria and archaea, respectively) and of treatments (*n* = 4) together with respective SD are shown. Table S3: Relative abundances of anaerobic fungal clades in the ITS1 clone libraries constructed from rumen samples of goats fed different diets (AH at d20 and HG at d30). Based on the study of Koetschan et al. (2014), the term clade is defined as a known species or an uncultivated subgroup (§) within a monophyletic lineage that has been identified using secondary structure-informed analysis of ITS1 region sequence data. Table S4: Part A lists all significant (*p* < 0.05) and positive (Pearson’s r > 0.8) correlation pairs of the investigated parameters for diet characteristics, NGS analysis of ITS2 and V4 and ITS2 clone library results. Only the lower triangle of the correlation matrix is shown. Part B lists all significant (*p* < 0.05) and negative (Pearson’s r < −0.8) correlation pairs of the investigated parameters for diet characteristics, NGS analysis of ITS2 and V4 and ITS2 clone library results. Only the lower triangle of the correlation matrix is shown. Below the total sum of correlation pairs for each parameter is exhibited. Figure S1: Rarefaction curve analysis of 16S rRNA gene amplicon sequences (V4 region; OTU-based) and the ITS2 region (ASV-based). Figure S2: Diversity analyses of 16S rRNA gene amplicon sequences (V4 region) on top and the ITS2 region (bottom) based on OUT/ASV-richness (i.e., species richness) and Shannon–Wiener index. Figure S3: Significant log2fold changes (DESeq2) of major fungal phyla (top) and Neocallimastigomycota ASVs (bottom) upon diet switch from forage (AH) to high grain (HG) diet. Figure S4: Phylogenetic relationships of anaerobic fungal LSU gene sequences generated from the sample G3AH inferred using the UPGMA method with bootstrap values from 1000 replications. The analysis involved a total of 45 nucleotide sequences (35 sequences generated from the G3AH sample, 9 sequences of the cultured genera of anaerobic fungi (*Piromyces* sp. MK775335, KY368606, and KY368602, *N. cameroonii* NG060329, KR920745, and MG992493, *F. austinii* MG584215, MG584217, and MG605676) plus 1 outgroup sequence.
