# Supplementary material for: The Effect of a High-Grain Diet on the Rumen Microbiome of Goats with a Special Focus on Anaerobic Fungi
Source: Microorganisms. 2021 Jan 12;9(1):157. doi: 10.3390/microorganisms9010157 (PMC7827659; doi:10.3390/microorganisms9010157)

**Supplementary Material**

**The effect of a high-grain diet on the rumen microbiome of goats with a special focus on anaerobic fungi**

**Katerina O. Fliegerova ^1*#^, Sabine M. Podmirseg ^2#^, Julia Vinzelj ^2#^ Diego J. Grilli ^3#^, Simona Kvasnova ^1^, Dagmar Schierova ^1^, Hana Sechovcova ^1^, Jakub Mrazek ^1^, Giuliana Siddi ^4^, Graciela N. Arenas ^3^ and Giuseppe Moniello ^4^**

^1^ Laboratory of Anaerobic Microbiology, Institute of Animal Physiology and Genetics, Czech Academy of Sciences, Prague, Czech Republic; [fliegerova@iapg.cas.cz](mailto:fliegerova@iapg.cas.cz); [kvasnova@iapg.cas.cz](mailto:kvasnova@iapg.cas.cz); [schierova@iapg.cas.cz](mailto:schierova@iapg.cas.cz); mrazek@iapg.cas.cz

^2^ Institute of Microbiology, University of Innsbruck, Innsbruck, Austria Affiliation; [Sabine.Podmirseg@uibk.ac.at](mailto:Sabine.Podmirseg@uibk.ac.at); Julia. Vinzelj@uibk.ac.at

^3^ Área de Microbiología, Facultad de Ciencias Médicas, Universidad Nacional de Cuyo, Mendoza, Argentina; [diegogrilli@yahoo.com.ar](mailto:diegogrilli@yahoo.com.ar); [gnarenas@yahoo.com.ar](mailto:gnarenas@yahoo.com.ar)

^4^ Department of Veterinary Medicine, University of Sassari, Sassari, Italy 2; [moniello@uniss.it](mailto:moniello@uniss.it); [giuliana.siddi@gmail.com](mailto:giuliana.siddi@gmail.com)

***** Correspondence: fliegerova@iapg.cas.cz; Tel.: +420267090504

**^#^** These authors contributed equally to this work

Table S1: Taxonomic composition of goat rumen liquid under different diets (AH at d20 and HG at d30) based on DNA amplicon sequencing targeting the ITS2 region (Fungi and Neocallimastigomycota) and the 16S rRNA gene V4 region (Archaea and Bacteria). Read abundance (for V4 at ≥ 1%) is given at phylum- and genus-level. Additionally, for each treatment mean values ± SD are shown (n=4). Anaerobic fungi results highlighted in grey.

|  |  | **G1_AH_** | **G2_AH_** | **G3_AH_** | **G4_AH_** | **G1_HG_** | **G2_HG_** | **G3_HG_** | **G4_HG_** | **Mean_AH_** | | | **Mean_HG_** | | | |  |
| --- | --- | --- | --- | --- | --- | --- | --- | --- | --- | --- | --- | --- | --- | --- | --- | --- | --- |
| **Fungi** | **Phylum-level** |  |  |  |  |  |  |  |  |  |  |  | |  |  |  | |
|  | Ascomycota | 42.7 | 45.3 | 35.5 | 9.9 | 7.0 | 13.0 | 1.5 | 1.0 | 34.1 ± 17.7^#^ | | | 4.9 ± 3.4^#^ | | | |  |
|  | Basidiomycota | 21.3 | 19.3 | 9.9 | 5.7 | 2.3 | 0.4 | 1.5 | 0.7 | 14.9 ± 8.8*^#^ | | | 1.3 ± 0.8*^#^ | | | |  |
|  | Mucoromycota | 0.04 | 0.04 | 0.00 | 0.00 | 0.00 | 0.00 | 0.00 | 0.00 | 0.02 ± 0.02 | | | 0.00 ± 0.00 | | | |  |
|  | Neocallimastigomycota | 33.6 | 31.4 | 48.7 | 82.7 | 82.6 | 72.5 | 96.0 | 96.0 | 47.7 ± 18.0*^#^ | | | 88.3 ± 18.9*^#^ | | | |  |
|  | Unclassified Fungi | 2.4 | 4.0 | 5.9 | 1.6 | 8.1 | 14.1 | 1.1 | 2.3 | 3.3 ± 1.2^#^ | | | 5.5 ± 3.7^#^ | | | |  |
| **Neocalli-mastigomycota** | **Genus-level** |  |  |  |  |  |  |  |  |  |  |  | |  |  |  | |
|  | *Feramyces* | 0.00 | 0.00 | 13.3 | 46.7 | 69.4 | 54.3 | 75.7 | 66.7 | 22.6 ± 14.1*^#^ | | | 68.7 ± 16.6*^#^ | | | |  |
|  | *Orpinomyces* | 31.4 | 30.0 | 3.9 | 5.9 | 1.7 | 5.5 | 4.3 | 2.7 | 15.4 ± 4.3 | | | 3.4 ± 1.1 | | | |  |
|  | *Piromyces* | 9.8 | 10.0 | 20.9 | 11.8 | 10.0 | 15.7 | 5.9 | 10.3 | 12.6 ± 2.2^#^ | | | 9.5 ± 0.8^#^ | | | |  |
|  | *Tahromyces* | 49.8 | 48.5 | 11.9 | 9.1 | 0.7 | 1.6 | 0.7 | 1.0 | 25.5 ± 6.5^#^ | | | 0.9 ± 0.1^#^ | | | |  |
|  | unclassified Neocallimastigomycota | 9.0 | 11.5 | 50.1 | 26.5 | 18.1 | 22.9 | 13.3 | 19.2 | 23.9 ± 7.5 | | | 17.5 ± 1.9 | | | |  |
| **Archaea** | **Genus-level** |  |  |  |  |  |  |  |  |  |  |  | |  |  |  | |
|  | Unclassified Thermoplasmatales | 1.6 | 8.5 | 5.8 | 26.5 | 15.1 | 25.5 | 18.3 | 33.3 | 10.6 ± 11* | | | 23.1 ± 8.11* | | | |  |
|  | *Candidatus Methanomethylophilus* | 3.3 | 1.7 | 0 | 0 | 0 | 0 | 0 | 3.3 | 1.2 ± 1.57 | | | 0.83 ± 1.67 | | | |  |
|  | *Methanobrevibacter* 1 | 42.6 | 27.1 | 13.5 | 5.9 | 7.5 | 7.8 | 8.5 | 6.7 | 22.3 ± 16.2 | | | 7.63 ± 0.74 | | | |  |
|  | *Methanobrevibacter* 2 | 31.1 | 33.9 | 59.6 | 63.2 | 73.6 | 64.7 | 67.6 | 43.3 | 47.0 ± 16.8 | | | 62.3 ± 13.2 | | | |  |
|  | *Methanosphaera* | 21.3 | 28.8 | 21.2 | 4.4 | 3.8 | 2.0 | 5.6 | 13.3 | 18.9 ± 10.3 | | | 6.18 ± 5 | | | |  |
| **Bacteria** | **Phylum-level** |  |  |  |  |  |  |  |  |  |  |  | |  |  |  | |
|  | Bacteroidetes | 43.9 | 43.5 | 34.3 | 52.0 | 63.6 | 57.6 | 46.8 | 54.9 | 43.4 ± 7.24* | | | 55.7 ± 6.94* | | | |  |
|  | Chloroflexi | 1.8 | 1.4 | 2.3 | 4.2 | 2.2 | 3.3 | 2.1 | 1.2 | 2.4 ± 1.25 | | | 2.2 ± 0.85 | | | |  |
|  | Fibrobacteres | 1.5 | 1.2 | 0.1 | 0.1 | 0 | 0 | 0.1 | 0 | 0.7 ± 0.74 | | | 0 ± 0.03 | | | |  |
|  | Firmicutes | 43.0 | 45.1 | 54.2 | 32.9 | 27.7 | 30.2 | 41.4 | 35.6 | 43.8 ± 8.74 | | | 33.7 ± 6.09 | | | |  |
|  | Saccharibacteria | 0.5 | 0.3 | 1.5 | 0.5 | 0.2 | 0.2 | 0.2 | 0.1 | 0.7 ± 0.55 | | | 0.2 ± 0.05 | | | |  |
|  | Spirochaetae | 3.3 | 3.0 | 1.5 | 2.0 | 2.1 | 3.6 | 2.7 | 3.2 | 2.5 ± 0.86 | | | 2.9 ± 0.63 | | | |  |
|  | Synergistetes | 1.3 | 1.5 | 4.1 | 4.6 | 2.4 | 2.5 | 3.6 | 1.6 | 2.9 ± 1.71 | | | 2.5 ± 0.85 | | | |  |
|  | Tenericutes | 2.4 | 1.6 | 0.1 | 0.4 | 1.1 | 0.7 | 1.2 | 1.1 | 1.1 ± 1.06 | | | 1.0 ± 0.24 | | | |  |
|  | Verrucomicrobia | 1.2 | 1.2 | 0.1 | 0.5 | 0 | 0.1 | 0.2 | 0.3 | 0.7 ± 0.51 | | | 0.2 ± 0.13 | | | |  |
|  | Other < 1% | 1.1 | 1.5 | 1.8 | 3.0 | 0.8 | 2.0 | 1.6 | 2.1 | 1.8 ± 0.82 | | | 1.6 ± 0.59 | | | |  |
| **Bacteria** | **Genus-level** |  |  |  |  |  |  |  |  |  |  |  | |  |  |  | |
|  | *Butyrivibrio 2* | 4.35 | 6.36 | 6.42 | 3.99 | 3.26 | 3.07 | 5.14 | 4.58 | 5.28 ± 1.11 | | | 4.01 ± 0.85 | | | |  |
|  | *Christensenellaceae R-7 group* | 10.91 | 9.83 | 11.35 | 5.39 | 5.46 | 6.58 | 9.65 | 6.62 | 9.37 ± 1.99 | | | 7.08 ± 1.29 | | | |  |
|  | *Lachnospira* | 2.27 | 1.95 | 3.05 | 0.51 | 0.69 | 0.56 | 1.21 | 0.74 | 1.94 ± 0.72 | | | 0.80 ± 0.20 | | | |  |
|  | *Lachnospiraceae genus 10* | 0.95 | 2.14 | 0.06 | 0.06 | 0.00 | 0.00 | 0.13 | 0.00 | 0.80 ± 0.74 | | | 0.03 ± 0.05 | | | |  |
|  | *Lachnospiraceae NK3A20 group* | 2.15 | 2.71 | 4.67 | 1.71 | 0.44 | 1.25 | 2.16 | 1.98 | 2.81 ± 0.93 | | | 1.46 ± 0.61 | | | |  |
|  | *Mogibacterium* | 0.38 | 0.32 | 2.01 | 0.19 | 0.06 | 0.31 | 0.51 | 0.37 | 0.72 ± 0.64 | | | 0.31 ± 0.13 | | | |  |
|  | *Prevotella 1* | 17.98 | 18.97 | 11.48 | 18.31 | 23.73 | 22.45 | 17.33 | 22.22 | 16.69 ± 2.60* | | | 21.43 ± 2.05* | | | |  |
|  | *Prevotellaceae UCG-003* | 4.35 | 3.40 | 2.27 | 5.32 | 5.34 | 3.07 | 4.19 | 5.07 | 3.84 ± 1.00 | | | 4.42 ± 0.79 | | | |  |
|  | *Quinella* | 0.00 | 0.00 | 3.83 | 3.68 | 0.94 | 1.32 | 3.11 | 1.55 | 1.88 ± 1.88 | | | 1.73 ± 0.69 | | | |  |
|  | *Rikenellaceae RC9 gut group* | 11.67 | 10.46 | 9.14 | 13.50 | 19.46 | 16.87 | 11.62 | 13.61 | 11.19 ± 1.39 | | | 15.39 ± 2.77 | | | |  |
|  | *Ruminococcaceae NK4A214 group* | 1.07 | 1.13 | 1.75 | 2.47 | 2.76 | 3.26 | 2.92 | 1.67 | 1.61 ± 0.50 | | | 2.65 ± 0.49 | | | |  |
|  | *Ruminococcaceae UCG-002* | 0.25 | 0.19 | 0.84 | 2.41 | 0.25 | 0.44 | 0.70 | 0.62 | 0.92 ± 0.74 | | | 0.50 ± 0.16 | | | |  |
|  | *Ruminococcaceae UCG-005* | 1.83 | 2.71 | 0.39 | 0.51 | 0.82 | 1.19 | 0.89 | 1.30 | 1.36 ± 0.91 | | | 1.05 ± 0.20 | | | |  |
|  | *Sphaerochaeta* | 0.32 | 0.25 | 0.39 | 0.38 | 2.20 | 3.39 | 2.48 | 2.72 | 0.33 ± 0.05 | | | 2.70 ± 0.36 | | | |  |
|  | *Succiniclasticum* | 0.95 | 1.83 | 2.46 | 0.70 | 0.63 | 0.38 | 1.02 | 1.24 | 1.48 ± 0.66 | | | 0.81 ± 0.31 | | | |  |
|  | *Treponema 2* | 3.09 | 2.65 | 1.23 | 1.71 | 0.00 | 0.25 | 0.38 | 0.31 | 2.17 ± 0.70 | | | 0.24 ± 0.12 | | | |  |
|  | *Unclassified* | 12.18 | 10.27 | 11.09 | 14.01 | 8.91 | 10.78 | 10.35 | 9.59 | 11.89 ± 1.21 | | | 9.91 ± 0.66 | | | |  |
|  | *Uncultured Rumen Bacterium* | 6.56 | 7.50 | 6.49 | 11.66 | 12.12 | 11.66 | 10.03 | 9.03 | 8.05 ± 1.80 | | | 10.71 ± 1.18 | | | |  |

significance between treatments shown at the p<0.05 from paired t-test (*) and DESeq2 analysis (^#^);

Table S2: Abundance of anaerobic fungi, bacteria and archaea in rumen liquid samples of goats fed different diets (AH at d20 and HG at d30) determined by qPCR based on ITS1 and 16S copy number per g fresh matter (FM). Mean values of each sample (technical replicates n=3 for anaerobic fungi and n=2 for bacteria and archaea, respectively) and of treatments (n=4) together with respective SD are shown.

| Target |  | G1_AH_ | G2_AH_ | G3_AH_ | G4_AH_ | G1_HG_ | G2_HG_ | G3_HG_ | G4_HG_ | Mean_AH_ | Mean_HG_ |
| --- | --- | --- | --- | --- | --- | --- | --- | --- | --- | --- | --- |
| Anaerobic fungi | gene copies  g^-1^ FM | 3.48 x 10^8^ | 3.84 x 10^8^ | 1.27 x 10^8^ | 1.31x 10^8^ | 3.87 x 10^7^ | 2.32 x 10^7^ | 5.43 x 10^8^ | 2.84 x 10^8^ | 2.47 x 10^8^ | 2.22 x 10^8^ |
|  | SD | 2.57 x 10^7^ | 2.84 x 10^7^ | 3.43 x 10^7^ | 1.83 x 10^7^ | 3.40 x 10^6^ | 2.20 x 10^6^ | 5.07 x 10^7^ | 3.08 x 10^7^ | 1.38 x 10^8^ | 2.45 x 10^8^ |
| Bacteria | gene copies  g^-1^ FM | 1.64 x 10^10^ | 1.60 x 10^10^ | 2.49 x 10^10^ | 2.59 x 10^10^ | 9.26 x 10^9^ | 1.80 x 10^10^ | 1.39 x 10^10^ | 3.09 x 10^10^ | 2.08 x 10^10^ | 1.80 x 10^10^ |
|  | SD | n.a. | n.a. | n.a. | n.a. | n.a. | n.a. | n.a. | n.a. | 5.36 x 10^9^ | 9.29 x 10^9^ |
| Archaea | gene copies  g^-1^ FM | 2.61 x 10^8^ | 3.28 x 10^8^ | 7.14 x 10^8^ | 5.63 x 10^8^ | 3.19 x 10^8^ | 4.57 x 10^8^ | 2.91 x 10^8^ | 9.06 x 10^8^ | 4.66 x 10^8^ | 4.93 x 10^8^ |
|  | SD | n.a. | n.a. | n.a. | n.a. | n.a. | n.a. | n.a. | n.a. | 2.1 x 10^8^ | 2.84 x 10^8^ |

Table S3: Relative abundances of anaerobic fungal clades in the ITS1 clone libraries constructed from rumen samples of goats fed different diets (AH at d20 and HG at d30). Based on the study of Koetschan et al. (2014), the term clade is defined as a known species or an uncultivated subgroup (§) within a monophyletic lineage that has been identified using secondary structure-informed analysis of ITS1 region sequence data.

| Genus | G1_AH_ | G2_AH_ | G3_AH_ | G4_AH_ | G1_HG_ | G2_HG_ | G3_HG_ | G4_HG_ | Mean_AH_ | Mean_HG_ |
| --- | --- | --- | --- | --- | --- | --- | --- | --- | --- | --- |
| *Feramyces* | 0 | 0 | 7.3 | 40.8 | 53.2 | 64.3 | 66 | 60.9 | 12 ± 19.5* | 61.1 ± 5.68* |
| *Joblinomyces* | 65.5 | 52.6 | 30.9 | 30.6 | 19.1 | 2.4 | 21.3 | 15.2 | 44.9 ± 17.2 | 14.5 ± 8.45 |
| JF423632 | 12.1 | 8.8 | 1.8 | 14.3 | 0 | 0 | 6.3 | 0 | 9.25 ± 5.46 | 1.58 ± 3.15 |
| *Piromyces* 4^§^ | 6.9 | 12.3 | 7.3 | 0 | 0 | 2.3 | 0 | 2.1 | 6.62 ± 5.05 | 1.1 ± 1.27 |
| *Tahromyces* | 3.4 | 3.5 | 0 | 0 | 0 | 0 | 0 | 2.2 | 1.73 ± 1.99 | 0.55 ± 1.10 |
| *BlackRhino^§^* | 12.1 | 22.8 | 52.7 | 14.3 | 27.7 | 31 | 6.4 | 19.6 | 25.5 ± 18.7 | 21.8 ± 11 |

Figure S1: Rarefaction-curve analysis of 16S rRNA gene amplicon sequences (V4 region; OTU-based) and the ITS2 region (ASV-based).


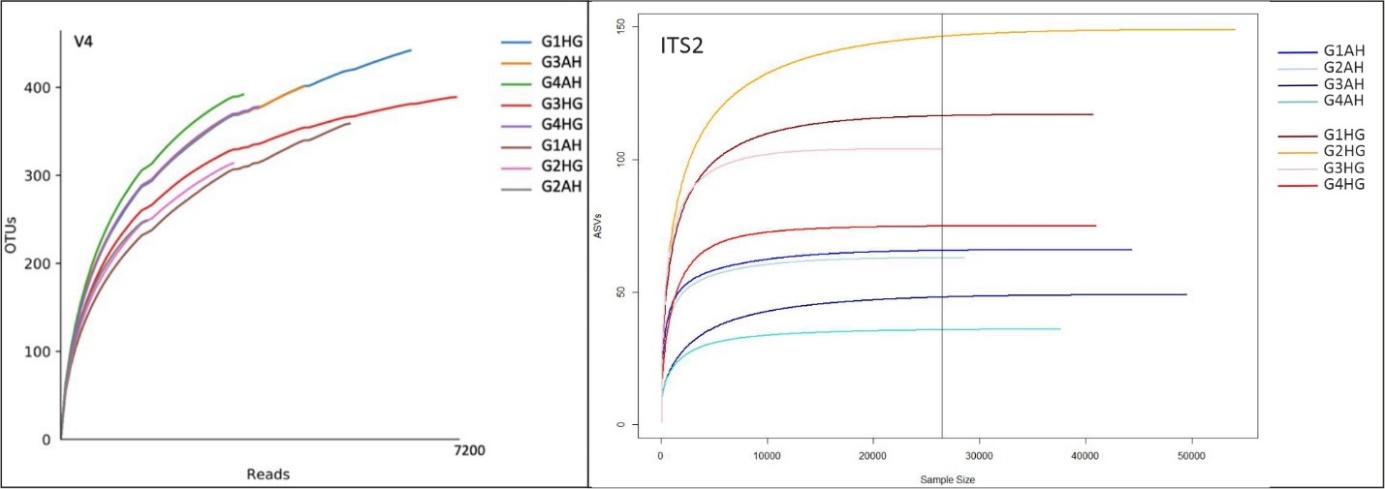


Figure S2: Diversity analyses of 16S rRNA gene amplicon sequences (V4 region) on top and the ITS2 region (bottom) based on OUT/ASV-richness (i.e. species richness) and Shannon-Wiener index.


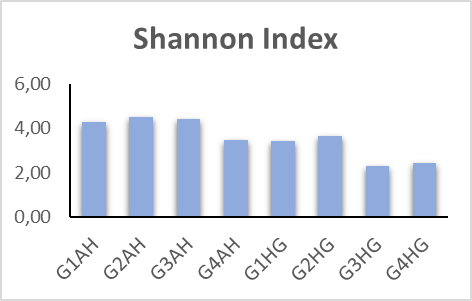

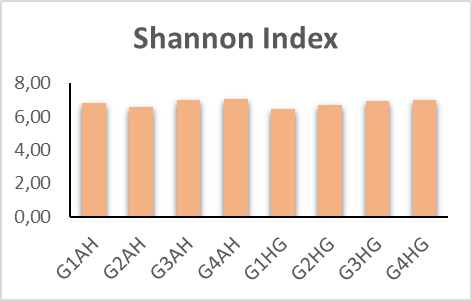

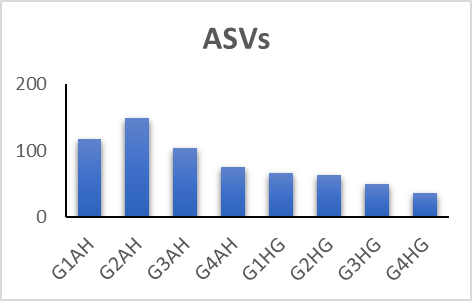

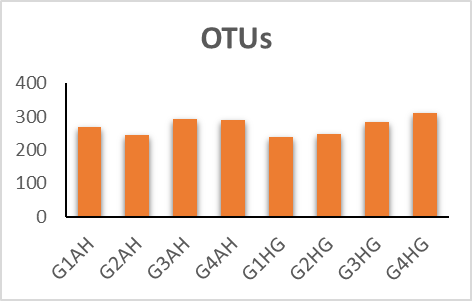


Figure S3: Significant log2fold changes (DESeq2) of major fungal phyla (top) and Neocallimastigomycota ASVs (bottom) upon diet switch from forage (AH) to high grain (HG) diet.

Figure S4: Phylogenetic relationships of anaerobic fungal LSU gene sequences generated from the sample G3AH inferred using the UPGMA method with bootstrap values from 1000 replications. The analysis involved a total of 45 nucleotide sequences (35 sequences generated from the G3AH sample, 9 sequences of the cultured genera of anaerobic fungi (*Piromyces* sp. MK775335, KY368606, and KY368602*, N. camerooni* NG060329, KR920745, and MG992493, *F. austinii* MG584215, MG584217, and MG605676) plus 1 outgroup sequence.


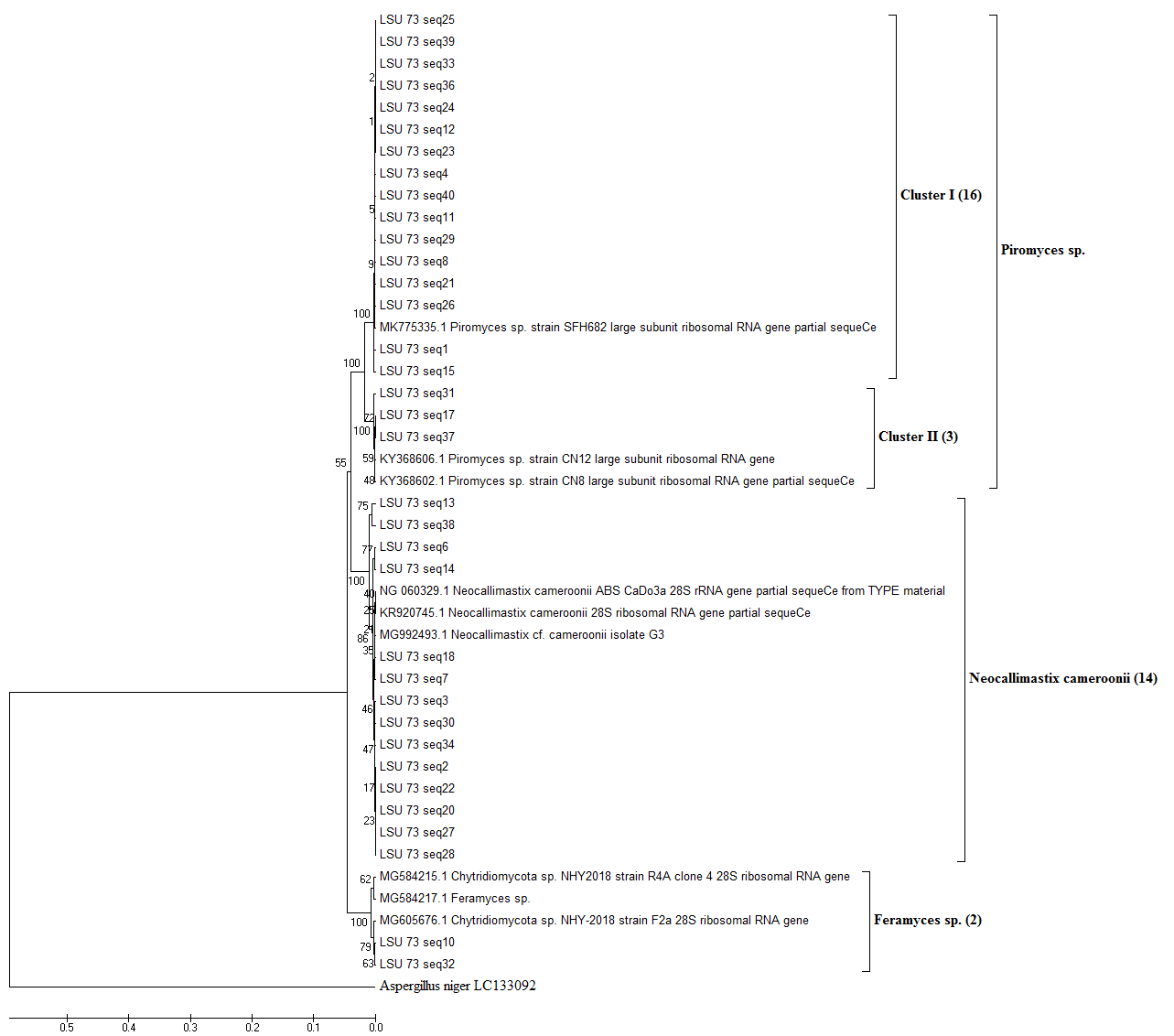

Supplement: Supplementary file 1 [file microorganisms-09-00157-s001.zip › Fliegerova et al. The effect of HG diet on goat rumen microbiom, Suppl. material/Supplementary Material Part A.docx]
